# *In planta* transcriptomics reveals conflicts between pattern-triggered immunity and the AlgU sigma factor regulon

**DOI:** 10.1371/journal.pone.0274009

**Published:** 2022-09-01

**Authors:** Haibi Wang, Amy Smith, Amelia Lovelace, Brian H. Kvitko

**Affiliations:** 1 Department of Plant Pathology, University of Georgia, Athens, Georgia, United States of America; 2 The Sainsbury Laboratory, Norwich Research Park, Norwich, United Kingdom; 3 The Plant Center, University of Georgia, Athens, Georgia, United States of America; Academia Sinica, TAIWAN

## Abstract

In previous work, we determined the transcriptomic impacts of flg22 pre-induced Pattern Triggered Immunity (PTI) in *Arabidopsis thaliana* on the pathogen *Pseudomonas syringae* pv. *tomato* DC3000 (*Pto*). During PTI exposure we observed expression patterns in *Pto* reminiscent of those previously observed in a *Pto algU* mutant. AlgU is a conserved extracytoplasmic function sigma factor which has been observed to regulate over 950 genes in *Pto* in growth media. We sought to identify the AlgU regulon when the bacteria are inside the plant host and which PTI-regulated genes overlapped with AlgU-regulated genes. In this study, we analyzed transcriptomic data from RNA-sequencing to identify the AlgU regulon (while in the host) and its relationship with PTI. Our results showed that the upregulation of 224 genes while inside the plant host require AlgU, while another 154 genes are downregulated dependent on AlgU in *Arabidopsis* during early infection. Both stress response and virulence-associated genes were upregulated in a manner dependent on AlgU, while the flagellar motility genes are downregulated in a manner dependent on AlgU. Under the pre-induced PTI condition, more than half of these AlgU-regulated genes have lost induction/suppression in contrast to mock treated plants, and almost all function groups regulated by AlgU were affected by PTI.

## Introduction

The plant cell produces surface receptors that allow it to recognize Pathogen Associated Molecular Patterns (PAMPs) and trigger Pattern Triggered Immunity (PTI). In the model organism *Arabidopsis thaliana* Col-0, PTI induction is associated with a series of responses including a rapid ROS (reactive oxygen species) burst, induction of defensive hormone biosynthesis pathways, changes in apoplast and cell wall composition, and increased expression of pathogen-recognition receptors [1–4]. The flagellin-derived peptide flg22 is a synthetic PAMP commonly used to activate PTI in laboratory settings. Upon recognizing flg22, the *Arabidopsis* FLS2 receptor initiates PTI responses, reducing proliferation of the model pathogen *Pseudomonas syringae* pv. *tomato* DC3000 (*Pto*) [5]. An activated PTI response also restricts Type III effector delivery by *Pto* and reduces expression of the *P*. *syringae* virulence regulon [6–9]. The early stages of infection are crucial as the pathogen and host compete for Type III Secretion System (T3SS) activation and restriction [6]. Bacteria rapidly change transcriptional profiles during early stages of infection [7], activating genes crucial for disease development [9, 10].

Upon transitioning into the leaf apoplast *Pto* has the capacity to perceive and respond to niche-specific cues to reprogram gene expression and express plant virulence factors. Extracytoplasmic function (ECF) sigma factors are a common tool used by bacteria to achieve such reprogramming. ECF sigma factors are kept inactive by their corresponding anti-sigma factors typically through direct protein-protein interaction. Upon signal perception, the anti-sigma factors are degraded and the ECF sigma factors are released and activated. One ECF sigma factor, AlgU (RpoE), is known to regulate hundreds of genes involved in metabolism, motility, stress tolerance, and virulence in *Pto* [11]. Deletion of the *algU* gene or the *algUmucAB* operon, which includes the anti-sigma factors of AlgU, reduces *Pto* growth in tomato and *Arabidopsis* seedlings [11, 12].

Previous studies have shown that motility related genes, osmotic stress response genes, and alginate synthesis genes in *Pto* are differentially expressed during early stage of infection in PTI pre-induced *Arabidopsis* compared to mock treated plants [7, 9]. Since these genes were also identified as part of the AlgU regulon [11, 13], we hypothesized that *Arabidopsis* PTI may disrupt AlgU-mediated regulation during early stages of *Pto* infection. However, the previously defined *Pto* AlgU regulon was determined by using an AlgU-overproduction strain in rich media [11], it is not known which genes belong to the AlgU regulon while in the plant host, under the native level of AlgU, and are specifically differentially expressed in the apoplastic-niche. In this study, we identified the *Pto* AlgU regulon while in the plant host during early infection, and confirmed that pre-induced PTI intervenes against the induction or suppression of more than half of the AlgU regulon (while in the host).

## Results

### Transcriptomic differences between Pto WT and ΔalgU during early *Arabidopsis* infection reveals the niche-specific AlgU regulon

**Fig 1:**
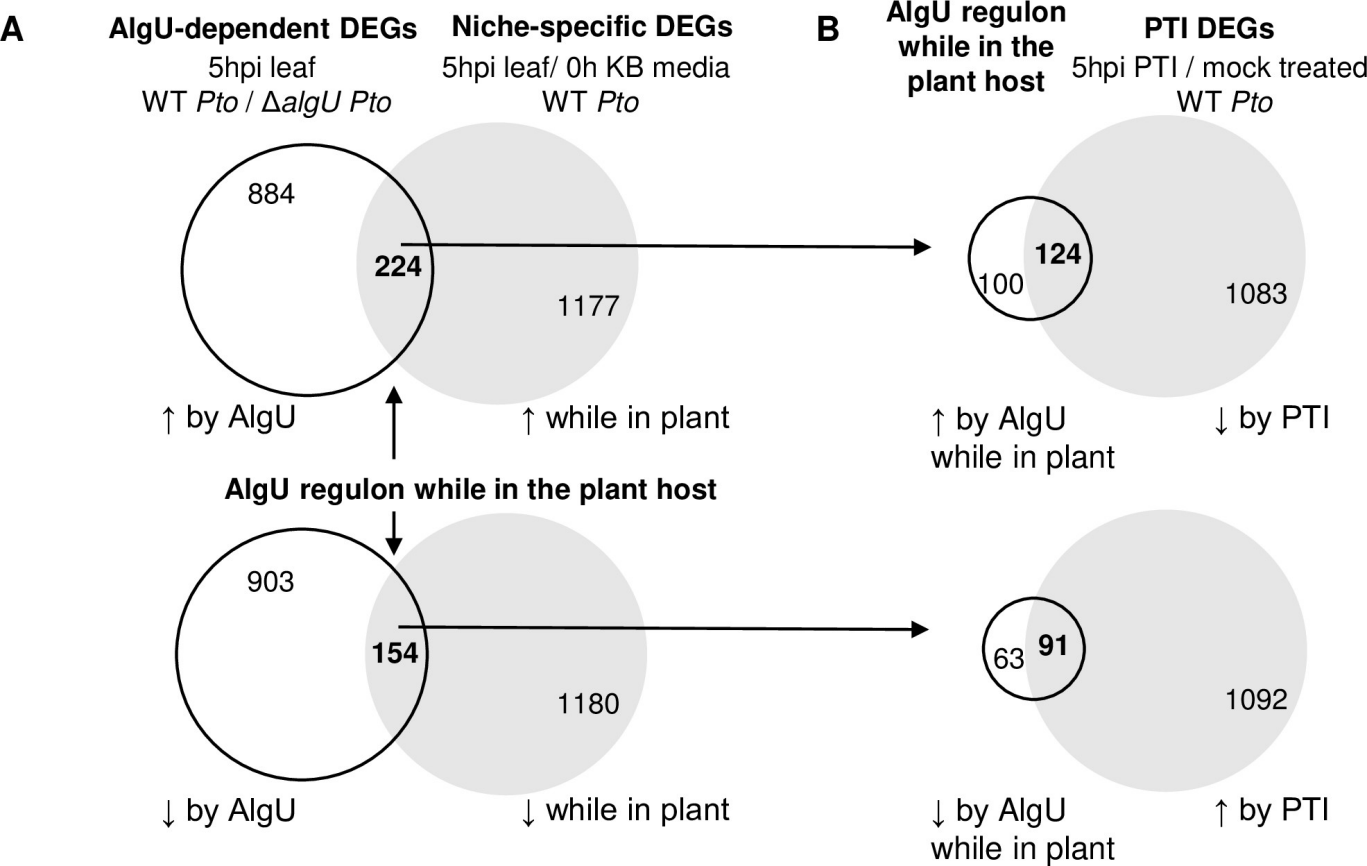
Venn diagrams showing the number of *Pto* differentially-expressed genes (DEGs) while in the plant host, AlgU-dependency, and overlap of the AlgU regulon (while in the host) with PTI-mediated regulation. **A**. The right grey circles indicate *Pto* DEGs in the *Arabidopsis* apoplast compared with KB growth media. The left white circles are AlgU-dependent DEGs. The overlap regions indicate *Pto* apoplast DEGs whose expression is dependent on AlgU, thus defining the AlgU regulon while in the plant host for the 5 h time point in *Arabidopsis*. **B**. The proportion of counter-regulated genes based on the overlap between the AlgU regulon (while in the host) (white circles) and with previously, identified *Pto* DEGs in the PTI pre-induced *Arabidopsis* apoplast (grey circles).

In order to identify the niche-specific AlgU regulon, we compared two sets of differentially-expressed *Pto* genes. The first set (Fig 1A right) contains *Pto* genes that are differentially expressed in the *Arabidopsis* apoplast at 5 hpi (hours post inoculation) compared to a time 0 *Pto* inoculum prepared from KB growth media. The genes in this set are either regulated by AlgU while in the plant host, or are regulated by other *Pto* transcription factors. The second set (Fig 1A left) of genes are those expressed differently in *ΔalgU* background compared to *Pto* wild type (WT) at 5 hpi in the *Arabidopsis* leaves, indicating that their expression is regulated by AlgU. The genes in this set include genes both specifically regulated while in the plant host as well as niche-independent AlgU-regulated genes.

To gather the transcriptomic data for the *in planta* sample, we infiltrated *Arabidopsis* with either WT or *ΔalgUmucAB* (*ΔalgU*) *Pto*, and collected RNA samples at 5 hpi for RNA-seq and data analysis using previously established techniques [7]. This was compared with RNA samples from the T0 *Pto* inoculum prepared from KB growth medium. By using a cutoff at 1.3-fold change in expression and false discovery rate less than 5%, we obtained the two sets of genes for comparison. The union of the two lists, which include both genes that are AlgU regulated and genes that are differentially expressed in a plant-specific manner, showed that the AlgU regulon (while in the host) include 224 AlgU-upregulated genes and 154 AlgU-downregulated genes (Fig 1A, S1 and S2 Tables in S2 File). To confirm and support the RNA-seq result, we also performed Reverse Transcription-quantitative PCR (RT-qPCR) on selected genes that belong to functional groups of interest, including alginate production (*algD*), osmotic stress response (*opuCA*), motility (*fliD*), and plant virulence (*avrPto1*) and obtained similar results (S1 Fig in S1 File). It is worth noting that since some of the genes in the AlgU regulon (while in the host) are arranged as polycistronic operons, the number of differently regulated promoters/transcripts regulated by AlgU will be smaller than the numbers of genes in the list.

For the AlgU upregulated regulon (while in the host), we were able to identify genes involved in osmotic stress response, oxidative stress response, alginate synthesis and export, cell shape and division pathways, DNA repair pathways, Type II Secretion System (T2SS) structural genes, macromolecule metabolism and energy production (including lipid metabolism, amino acid metabolism, nucleotide metabolism, tRNA synthesis, ribosomal protein, transporter and permease genes). We also confirmed previous observations that a subset of T3SS effector genes and transcription factors are part of the AlgU regulon while in the plant host (Table 1).

**Table 1:** Functional groups of AlgU up-regulated genes while in the plant host.

Number of genes	Function
36	Hypothetical and unknown function
26	Transporter, permease, and lipoprotein
23	Amino acid, nucleotide, lipid metabolism, and energy production
19	T3SS related
19	Transcription regulator
16	tRNA
14	Protein translation and folding
13	Alginate synthesis
10	Ribosomal protein
9	Osmotic stress
6	T2SS structural
6	Oxidative response
5	Cell shape maintenance and division
5	DNA repair
4	Coronatine synthesis
3	Phage related
2	Iron-sulfur cluster related
2	Other secretion system
2	SOS response
2	Antibiotic resistance and toxin/antitoxin
2	Plasmid mobility gene

For the AlgU downregulated regulon (while in the host), we identified genes involved in flagellar assembly, chemotaxis, signal transduction pathways, Type IV conjugation pilus assembly, DNA modification pathways, and macromolecule metabolism and energy production (including lipid metabolism, amino acid metabolism, nucleotide metabolism, protein translation and degradation, and transporter genes). We also identified GGDEF/EAL domain containing proteins and transcription factors among downregulated genes (Table 2).

**Table 2:** Functional groups of AlgU down-regulated genes while in the plant host.

Number of genes	Function
35	Hypothetical and unknown function
32	Amino acid, nucleotide, and lipid metabolism, and energy production
14	Transporter, permease, and lipoprotein
13	Transcription regulator
11	Flagella related
10	Chemotaxis
10	Receptor and signal transduction
8	Conjugal transfer protein
6	Protein translation, folding, and protease
5	Phage or transposase
3	GGDEF domain containing protein
3	DNA modification
3	Sulfur metablism or iron-sulfur cluster related
1	Antibiotic synthesis

### Pre-induced PTI response intervenes against the niche-specific AlgU regulon

It was previously observed that the *Pto* genes affected by pre-induced PTI include those that had been observed to be regulated by AlgU. To further identify the portion of the niche-specific AlgU regulon that is affected by PTI, we compared the AlgU regulon to the list of genes that are affected by PTI [7]. We found that 124 (55%) genes from the AlgU upregulated regulon (while in the host), and 91 (59%) genes from the AlgU downregulated regulon are intervened against by the pre-induced PTI (Fig 1B), which involves all the functional groups except iron-sulfur cluster related genes (Table 3, S3, and S4 Tables in S2 File). For the majority of the remaining genes, namely 86 out of these 100 genes from the AlgU upregulated regulon, and all 63 from the AlgU downregulated regulon remain the similar expression level under pre-induced PTI conditions compare to mock treated conditions; while in contrast, 14 out of these 100 genes in the AlgU upregulated regulon are further upregulated under pre-induced PTI condition, and none of the AlgU-suppressed regulon genes is further downregulated (S5 Table in S2 File). Taken together, these numbers suggest that the pre-induced PTI response and AlgU regulon are in conflict.

**Table 3:** PTI intervenes most AlgU-regulated functional groups.

AlgU induced genes while in the plant host:		
PTI intervene percentage of the AlgU niche-specific regulon^a^	Number of genes	Function
61.54%	16	Transporter, permease, and lipoprotein
100.00%	13	Alginate synthesis
36.11%	13	Hypothetical and unknown function
52.17%	12	Amino acid, nucleotide, lipid metabolism, and energy production
57.89%	11	T3SS related
78.57%	11	Protein translation and folding
90.00%	9	Ribosomal protein
47.37%	9	Transcription regulator
88.89%	8	Osmotic stress
100.00%	4	Coronatine synthesis
50.00%	3	T2SS structural
100.00%	3	Phage related
40.00%	2	Cell shape maintenance and division
12.50%	2	tRNA
100.00%	2	Other secretion system
40.00%	2	DNA repair
33.33%	2	Oxidative response
50.00%	1	Antibiotic resistance and toxin/antitoxin
50.00%	1	Plasmid mobility gene
0.00%	0	Iron-sulfur cluster related
0.00%	0	SOS response
AlgU down-regulated genes:		
PTI intervene percentage of the AlgU niche-specific regulon^a^	Number of genes	Function
53.13%	17	Amino acid, nucleotide, and lipid metabolism, and energy production
45.71%	16	Hypothetical and unknown function
90.91%	10	Flagella related
71.43%	10	Transporter, permease, and lipoprotein
76.92%	10	Transcription regulator
90.00%	9	Chemotaxis
70.00%	7	Receptor and signal transduction
50.00%	3	Protein translation, folding, and protease
66.67%	2	GGDEF domain containing protein
40.00%	2	Phage or transposase
66.67%	2	Sulfur metabolism or iron-sulfur cluster related
100.00%	1	Antibiotic synthesis
12.50%	1	Conjugal transfer protein
33.33%	1	DNA modification

a. Percent of genes in each function groups in Tables 1 or 2 that are intervened by PTI.

### Stress responsive genes are AlgU induced and intervened against by PTI at 5 hpi

**Fig 2:**
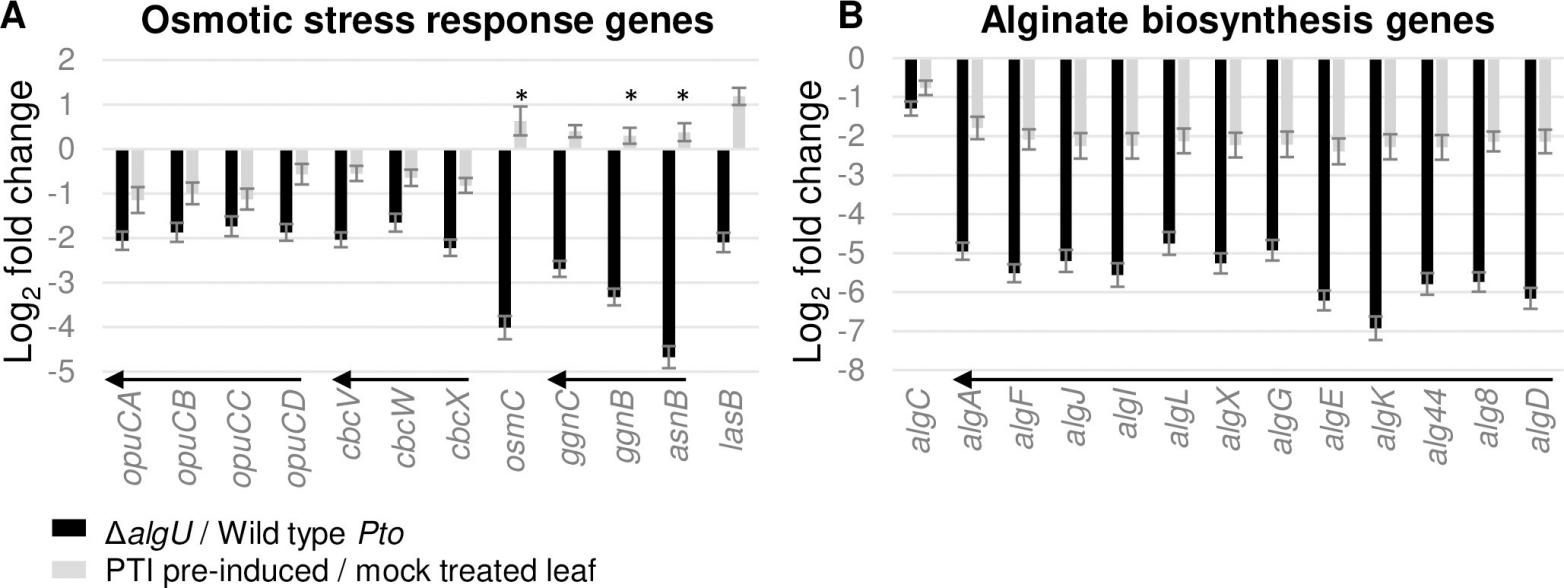
Expression changes of stress response related genes. **A**. Osmotic stress response genes. **B**. Alginate synthesis genes. * indicates genes with padj >0.05 calculated by DESeq2. All * in this graph are from grey bars. Arrows indicate genes within an operon.

AlgU regulates the expression of osmotic and oxidative stress response genes in different bacteria [14]. Alginate, a secreted polysaccharide, is also generally considered to shield bacteria from external stressors [15, 16]. We analyzed the relationship between AlgU and these stress tolerance related genes while in the plant host. Glycine betaine transporter genes (*opuCABCD*, *cbcVWX*) [17, 18] were dependent on AlgU for induction, and are PTI inhibited (Fig 2A). However, for compatible solute synthesis genes, even though their induction is AlgU dependent, their expression was not inhibited by the pre-induced PTI at 5 hpi. The oxidative stress response genes [13] *trx-2* (PSPTO_5243), *sodB* (PSPTO_4363), and a glutaredoxin domain protein (PSPTO_4161) are also part of the AlgU regulon (while in the host) that are intervened against by PTI (S1 and S3 Tables in S2 File). Alginate synthesis genes showed a similar trend to the glycine betaine transporter genes, they are induced by AlgU, and intervened against by pre-induced PTI (Fig 2B). These results suggest that at 5 hpi, the bacterial cells activate genes related to osmotic stress response and alginate production with the help of AlgU, while pre-induced PTI can have a negative effect on the induction, which likely reduces the bacterial capability for tolerating stresses.

### Multiple secretion system-associated genes are AlgU induced and intervened against by PTI at 5 hpi

**Fig 3:**
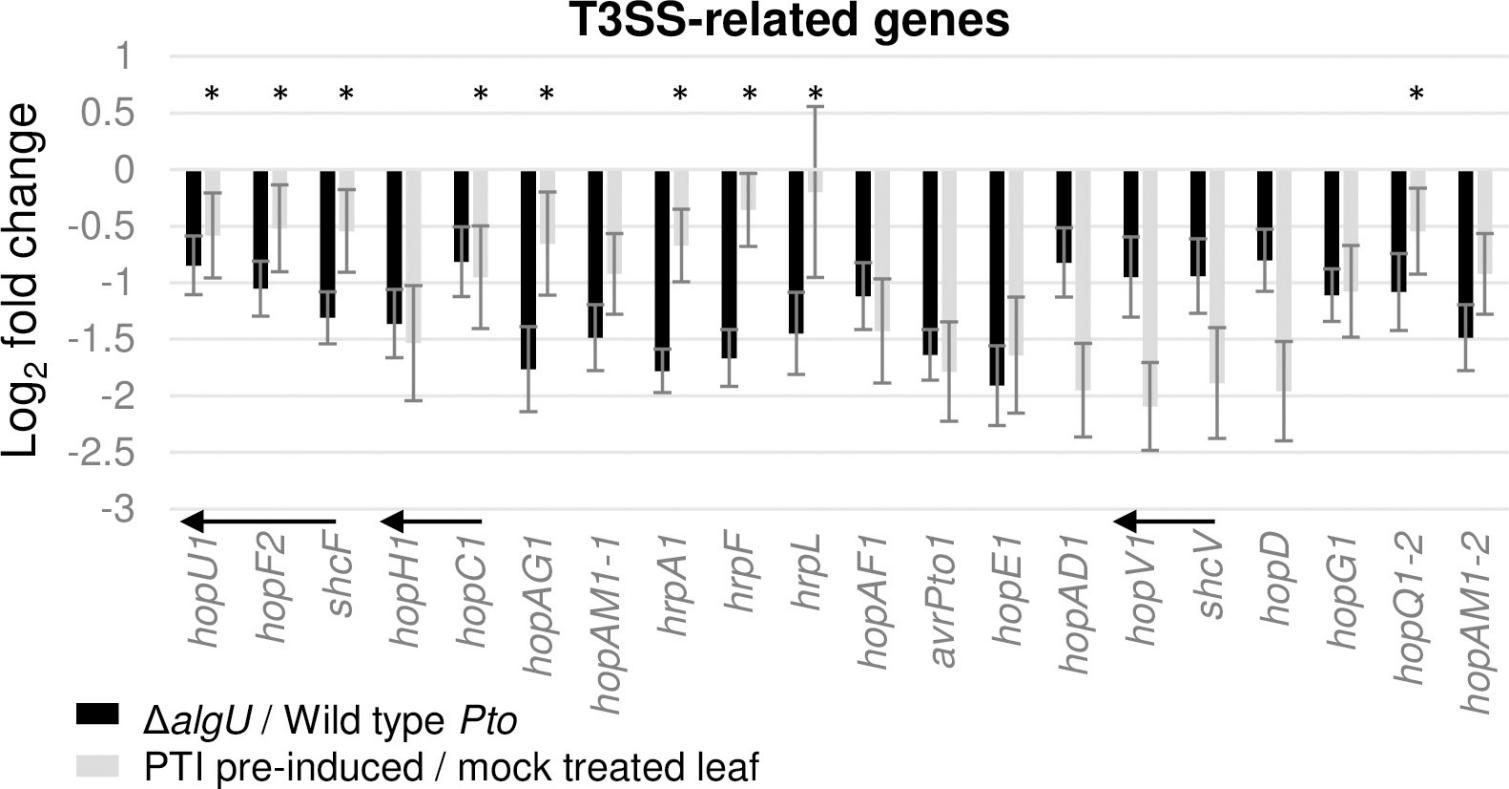
Expression changes of Type III Effectors and Type III Secretion System (T3SS) related genes that were identified as AlgU regulated in this study. * indicates genes with padj >0.05 calculated by DESeq2. All * in this graph are from grey bars. Arrows indicate genes within an operon.

The T3SS is a needle-like structure that delivers effector proteins to the plant cell cytoplasm, and is required for *Pto* virulence [8, 19]. Type III effectors (T3Es) are critical for suppressing PTI responses in mock treated plants. Genes encoding T3Es are distributed throughout the *Pto* genome [20, 21]. Our data showed that AlgU enhances induction of 17 out of the 36 T3Es at 5 hpi, and these same genes are suppressed by pre-induced PTI (Fig 3). However, T3SS structural genes are not significantly AlgU induced, even though they are suppressed by pre-induced PTI (S2 Fig in S1 File).

**Fig 4:**
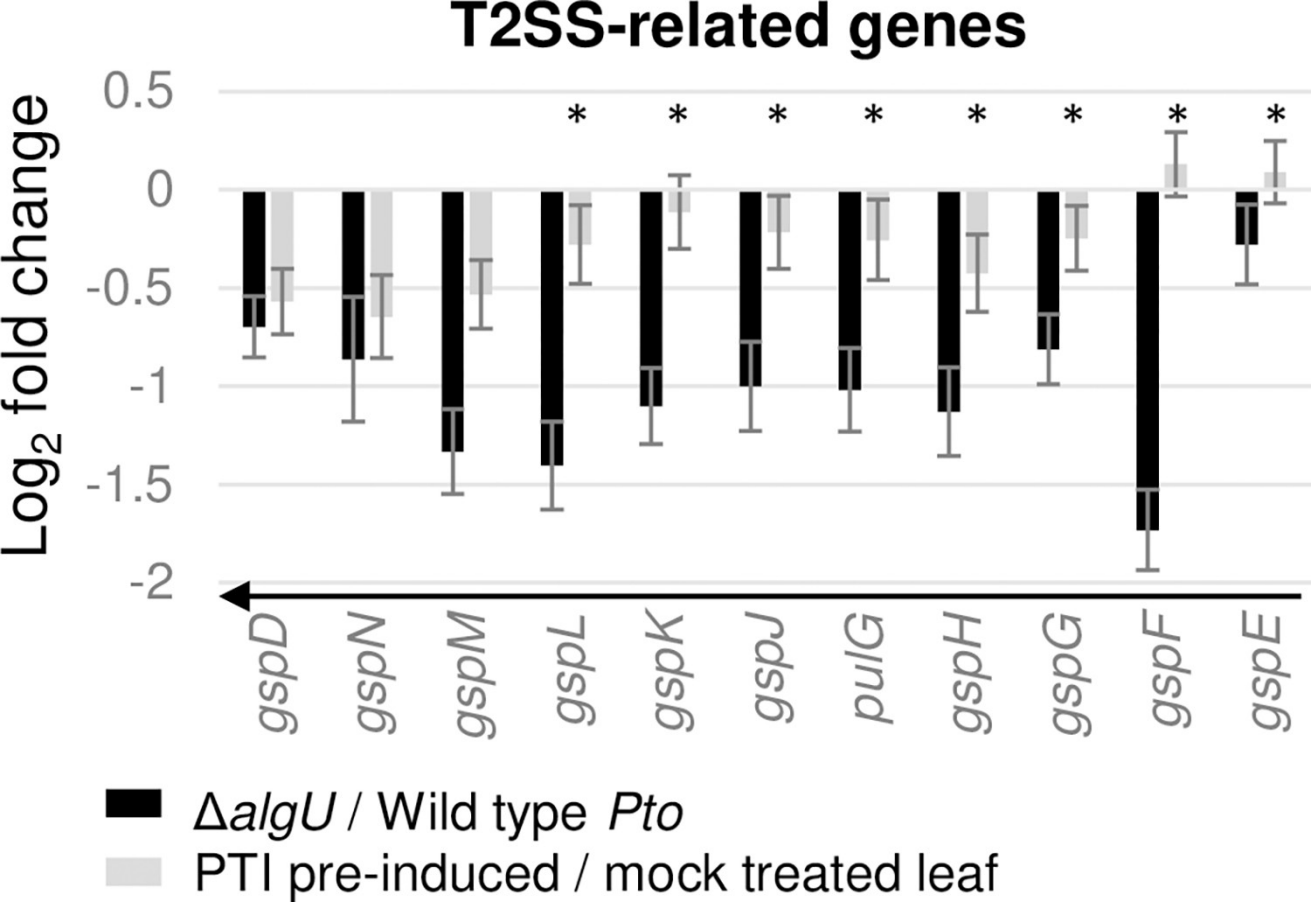
Expression changes of Type II Secretion System (T2SS) pathway genes. * indicates genes with padj >0.05 calculated by DESeq2. All * in this graph are from grey bars. Arrows indicate genes within an operon.

Another secretion system that may also play a role in PTI response manipulation, the Type II Secretion System (T2SS), transports folded proteins from periplasm to the extracellular space. Our data showed that ten T2SS structural component genes are AlgU induced at 5 hpi, and *gspD*, *gspN*, and *gspM* are suppressed in the pre-induced PTI environment (Fig 4). Interestingly, *gspDNM* are the three last genes within the cluster, and the first several genes in the same cluster are not significantly PTI-suppressed. One explanation is that these genes may be transcribed from separate promoters.

Coronatine is a plant hormone mimic and virulence factor produced by *Pto*. It was shown previously that AlgU may play a role in coronatine synthesis gene regulation [12]. Our data showed that, at 5 hpi, AlgU significantly upregulated the expression of only two coronatine-associated genes: *hopAQ1*, which may be co-transcribed with the regulator for coronafacic acid (CFA) and coronamic acid synthesis (CMA) called *corRS*, but is not known to directly relate to coronatine synthesis, and *cmaL*, which is encoded away from the primary biosynthetic CFA and CMA operons [22] (S3 Fig in S1 File). Regardless of the limited involvement of AlgU in coronatine gene regulation, most of the coronatine biosynthesis genes are inhibited by pre-induced PTI.

### Flagellar motility-related genes are AlgU suppressed and intervened against by PTI at 5 hpi

**Fig 5:**
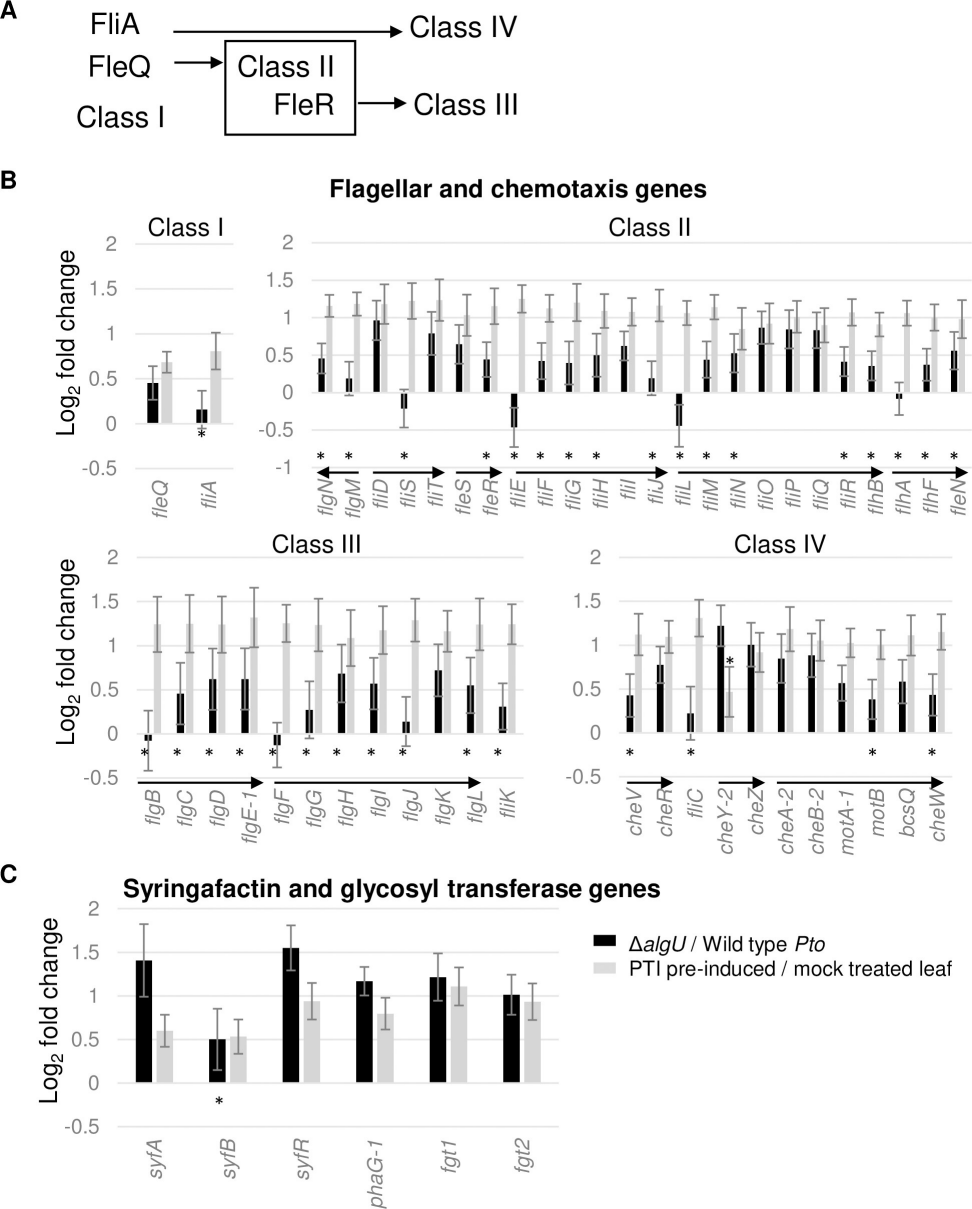
Expression changes of motility genes. **A**. Sketch showing the four classes belonging to the motility gene regulatory hierarchy. **B**. Log_2_ fold change of the motility genes organized by classes. **C**. Genes related to swarming motility (syringafactin) and flagella glycosylation. * indicates genes with padj >0.05 calculated by DESeq2. * for black bars are placed under line of 0, * for grey bars are placed above line of 0. Arrows indicate genes within an operon.

In rich media, AlgU suppresses swimming motility by lowering the expression of several flagella-related genes including the *fliC* flagellin gene. Reduced flagellin expression can reduce FLS2-mediated responses in tobacco and tomato [23]. We sought to determine how AlgU regulates motility related genes in *Arabidopsis*. The 60+ flagellar assembly and chemotaxis related genes in *Pto* are organized as a large single gene cluster on the chromosome. Studies in *P*. *aeruginosa*, which possesses a syntenous gene cluster, suggested that these genes can be organized into four classes based on their expression hierarchy [24] (Fig 5A). Our data showed that the presence of AlgU in the WT background reduced expression of most of the four classes of genes at 5 hpi, and PTI-exposure increased the expression of all these genes (Fig 5B).

Interestingly, several genes proposed to be involved in swarming motility showed the same trend as the four classes of flagellar genes (Fig 5C). Syringafactin and 3-(3-hydroxyalkanoyloxy) alkanoic acid (HAA) are two surfactants, and their production is directly related to swarming motility [25, 26]. The syringafactin production gene *syfA* and its transcription regulator *syrR* (also called *syfR*), and the HAA production gene *rhlA* homolog *phaG-1* (PSPTO_3299) are all downregulated by AlgU and upregulated under pre-induced PTI conditions. In addition, the two genes *fgt1* and *fgt2* (PSPTO_1946/1947), which contribute to flagellar glycosylation and swarming motility [27], are similarly regulated. These results suggest that AlgU promotes the cells to enter a non-motile state at 5 hpi, while pre-induce PTI promotes the cells to keep expressing motility related genes.

### Multiple transcription factors have altered regulation while in the plant host in the absence of AlgU, and are affected against by pre-induced PTI at 5 hpi

**Fig 6:**
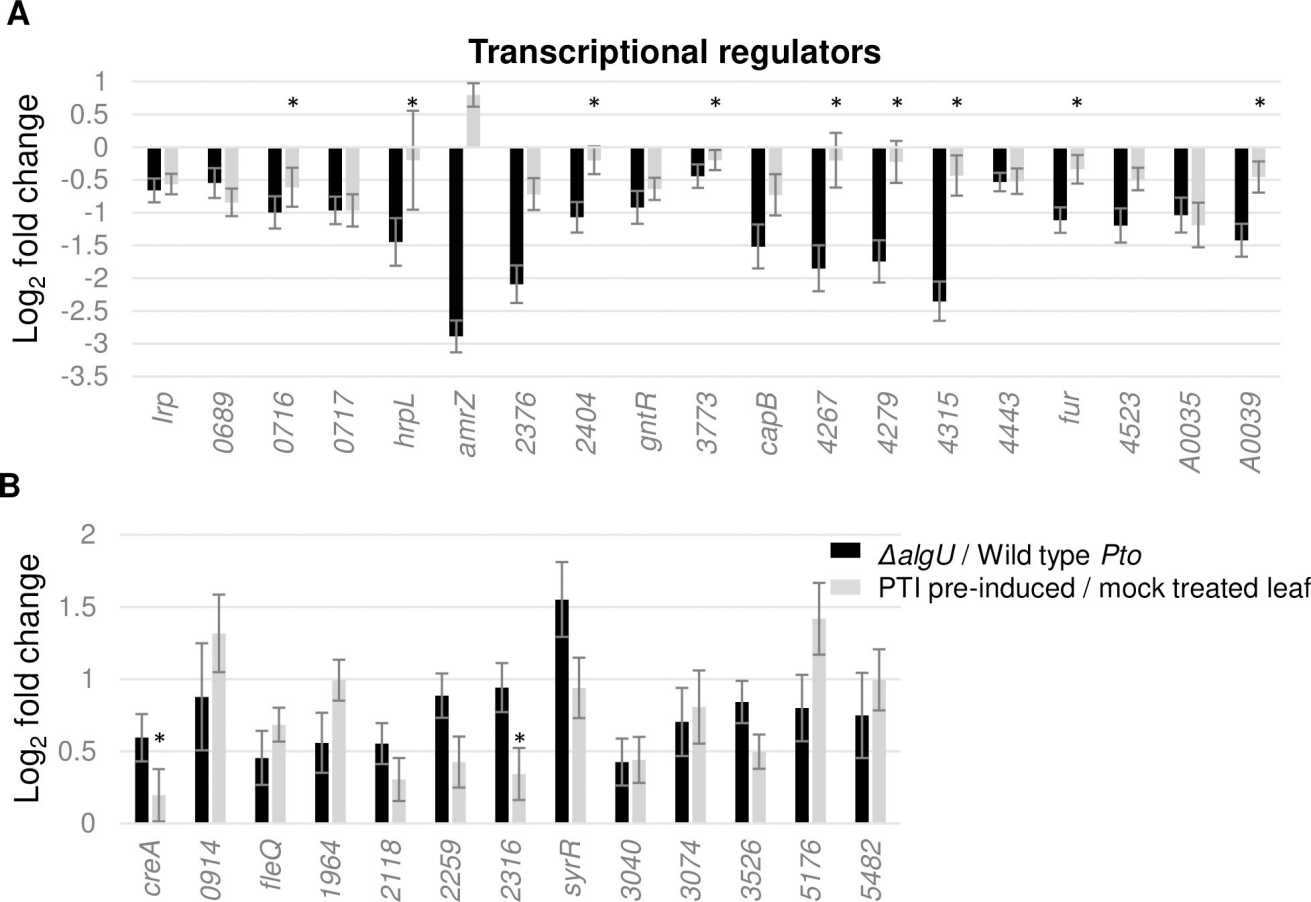
Expression changes of transcriptional regulators that are differentially-expressed in the absence of AlgU in mock treated plant. **A**. Genes downregulated in *ΔalgU* background. **B**. Genes upregulated in *ΔalgU* background. * indicates genes with padj >0.05 calculated by DESeq2. All * in this graph are from grey bars.

Previous ChIP-Seq analysis has shown that many of the AlgU-regulated genes have no indication of AlgU binding at their promoter [11]. One explanation is that AlgU affects the expression of these genes indirectly through other transcriptional regulators, either by direct regulation, or through other regulatory feedbacks. Our data showed that 19 genes with known or predicted transcriptional regulatory function, including *hrpL*, *amrZ*, and *fur* are induced when AlgU is present, and another 13 transcriptional regulators including *fleQ* and *syrR* are suppressed in the presence of AlgU at 5 hpi (Fig 6). Pre-induced PTI significantly affected 9 of these induced genes and 10 of these suppressed genes. Interestingly, *amrZ* showed a strong AlgU dependence for induction, but it is induced under PTI condition rather than suppressed, dissimilar to the others.

## Discussion

In this study, we defined the AlgU plant niche-specific regulon by comparing the *Pto* transcriptome 5 hpi after inoculation into the *Arabidopsis* apoplast with the *Pto* DC3000 transcriptome with and without AlgU at the same time point. We focused on early infection stage (5 hpi) since this time period has been previously identified as a crucial stage for establishing the host pathogen relationship. We then compared the AlgU regulon (while in the host) with patterns of *Pto* gene regulation experienced under pre-induced PTI. We found that PTI exposure counter-regulates more than half of AlgU-regulated genes, and that PTI affects almost all functional groups regulated by AlgU. Our result showed that both PTI and AlgU impact pathways including stress tolerance, motility, and T3SS-associated genes. While we cannot confidently infer that PTI interferes directly with the function of AlgU, the large overlap in counter-regulated genes is striking. With the notable exception of *amrZ*, all genes shared by in the AlgU in plant regulon or in response to pre-induced PTI show contrasting patterns of regulation.

### AlgU plant niche-specific regulon is smaller from that was observed in growth media

While we identified over 2,000 genes differentially regulated by AlgU (>1.3 fold or <0.7 fold). However, the identified AlgU regulon specific to while in the plant host is significantly smaller, representing 224 upregulated and 154 downregulated genes. The smaller size of the AlgU regulon (while in the host) has also been observed by Yu et al. [13] which examined the AlgU regulon of *P*. *syringae* pv *syringae* B728a (*Psy* B728a) under different stressors in growth media or in association with plant hosts and showed that subsets of many transcription factor regulons are condition specific. In the plant niche, a subset of the transcriptional factors in the AlgU regulon likely incorporate condition specific signals to modulate and fine tune global gene expression patterns. In addition, many AlgU regulon genes are co-regulated by other transcription factors. Previous studies and our data have shown that the AlgU regulon overlaps with those of other transcription regulators such as the sigma factors RpoS, RpoN, HrpL, [11, 13], small RNA regulators RsmA2/A3 [28], the Fur iron homeostasis regulator [9, 29], and the two-component system CvsSR [30].

### Secretion systems

Consistent with the AlgU regulon in growth media, we have identified multiple T3Es as part of the AlgU regulon (while in the host). The HrpL sigma factor drives the expression of T3Es and HrpL itself shows reduced expression in the absence of AlgU [11, 13]. However, only a subset of T3Es show differential AlgU-mediated regulation. It is unclear why only a subset of T3E genes would show enhanced AlgU-dependent induction, when the simple prediction would be that reduced HrpL expression would have downstream effects on all HrpL-regulated T3Es. The AlgU-responsive T3E are from different effectors clusters on the genome, different identified functions in the plant cell. Some T3Es targets both PTI and Effector Triggered Immunity (ETI) pathways (HopAD1 [21] and HopU1 [31, 32]). Some T3Es are associated with cytoskeleton (HopG1 [33, 34] and HopE1 [35]). Some T3Es are associated with plant membrane proteins (HopF2 [36] and HopAF1 [37]). The only shared feature of these T3Es is that they suppress PTI. However, PTI suppression is a common feature of many T3Es that were not identified in the AlgU regulon (while in the host).

Unlike the T3SS, the potential role of T2SS in the host-pathogen relationship has been largely overlooked in *Pto*. In agreement with the previously published AlgU regulon in growth media [11], our data also showed that T2SS structural genes are upregulated by AlgU while in the plant host, and these genes are PTI suppressed. The T2SS, also known as the general secretion pathway (GSP), transports folded proteins across the outer membrane. It was previously observed that *Pto Δ*g*spD* and *ΔgspE* mutant showed significant reduction in disease development [38]. In contrast to *Pto*, T2SS is better understood in several other pathogens. In the necrotrophic pathogens *Pectobacterium atrosepticum* and *Dickeya dadantii*, T2SS is important for virulence, ROS tolerance [39, 40], and promotes commensal bacterial growth via secretion of pectate lyases and other cell-wall-degrading enzymes [41]. Similarly, in the root commensal bacteria *Dyella japonica*, T2SS plays a role in PTI suppression [42]. In the human pathogen *P*. *aeruginosa*, T2SS is involved in biofilm formation [43] and toxin secretion [44]. On the other hand, even though our data showed that T2SS is AlgU induced while in the plant hostand is PTI-suppressed, we do not know which *Pto* proteins are T2SS substrates, because the substrate recognition signal is not universal and cannot be easily predicted [45, 46]. The two known substrates PlcA1 (PSPTO_3648) and PlcA2 (PSPTO_B0005) [38] are neither significantly regulated by AlgU nor affected by pre-induced PTI (S6 and S8 Tables in S2 File). Based on research from other bacteria, the substrates secreted by T2SS in *Pto* may include lipases, proteases, phosphatases [47–49].

Interestingly, in contrast to a previous study which identified T6SS genes as a part of the *Psy* B728a AlgU regulon in the bean apoplast at 2 days post inoculation [13], we did not identify any T6SS genes. There are two clusters of T6SS genes in *Pto*, the HSI-I coded by genes PSPTO_2538–2554, and the HSI-II coded by PSPTO_5415–5438 [50]. Both encode structural genes and secreted factors. Our data did not identify any of these genes as part of the AlgU regulon (while in the host) (S6-S8 Tables in S2 File). This is similar to the previous observation that *Psy* B728a and *Pto* have different T6SS regulation in growth media (Freeman 2013), possibly because of major differences in life styles. *Psy* B728a is a broad-host-range pathogen and is a well-adapted epiphyte, with a small effector complement making large use of a cohort of toxins. *Pto* is a narrow host range pathogen and a poor epiphyte, with a large effector complement and the coronatine phytohormone mimic.

The difference between our result and the *Psy* B728a result may also because the *Psy* B728a AlgU regulon was sampled in bean leaves at 48 hours post infection. It has been demonstrated that the late infection stage transcriptome does not corelate well to genes that are crucial during the development of infection [51], while the early timepoint (>6 h) transcriptomic patterns can be used to predict the outcome of the host-pathogen interactions [9, 10].

### Motility genes

It is interesting that we saw both swimming and swarming motility/surfactant related genes are suppressed by AlgU at 5 hpi, and are upregulated under the pre-induced PTI condition. This suggests that, in addition to the previously proposed role for AlgU in immunity evasion, *Pto* may use AlgU-mediated regulation to adopt a nonmotile lifestyle in naïve plants. The enhanced expression of flagellar genes in the absence of AlgU has been shown to enhance immunity detection [23]. It is interesting that exposure to PTI-conditions results in a similar pattern of enhanced flagellar gene expression by *Pto* which would presumably also enhance immune detection by the host. It is unclear whether this is a maladaptive response by the bacteria that favors the host or if PTI-associated signals are perceived by the bacteria as repellants resulting in maintenance of motility and an avoidance response.

According to the flagellar gene regulatory hierarchy, the two transcription factors FleQ and FliA belong to class I. FleQ activates class II genes, which include the cytoplasmic structural genes and the regulator FleR. FleR then activates class III genes, which include the structural genes localized at the cell wall, outer membrane, and extracellular space. FliA activates class IV genes, which include flagellin, motor genes and chemotaxis genes [24] (Fig 5A). Interestingly, we observed two unexpected patterns from the RNA-seq data. First, although PTI exposure universally resulted in increased expression of all these genes, the first gene in all FleQ and FleR induced operons showed distinctive AlgU expression pattern in contrast to the other genes, as the first genes are either mildly induced or show no significant different while the following genes are consistently downregulated by AlgU. Additionally, FleQ was hypothesized to suppresses the expression of the syringafactin regulator *syrR* [25], but they both were suppressed by AlgU according to data from this study. These observations suggest that motility genes are not always co-regulated, and additional regulatory mechanisms may exist. Second, *fliA* expression level was not significantly increased in the absence of AlgU, but most of FliA-regulated genes increased expression regardless. This may be because the change in expression level in the transcription regulator is not in proportion to the change in its regulated genes. Surprisingly, our RNA-seq data did not show a significant increase in the expression of *fliC* (Class IV) in the strain without *algU*, which is contradictory to the previously reported results based on RT-qPCR using tomato as the host at 6hpi [23]. Instead, our data agrees with data from another group that also used *Arabidopsis* as the host plant [12]. Whether the difference in *fliC* expression is due to differences in the host environment or other experimental factors remains an open question.

### Pre-induced PTI and AlgU in conflict

Overall, our data indicates that the patterns of induction or suppression for AlgU-regulated genes while in the plant host are generally reversed during PTI exposure. At this point we are unable to conclude whether PTI-associated responses interfere with AlgU-mediated regulation directly or indirectly. The amount of free AlgU in the cell is post-translationally regulated by the anti-sigma factors MucA and MucB, which is regulated through the regulated intramembrane proteolysis (RIP) pathway. It was previously shown in *Arabidopsis* that two secreted plant proteases, SAP1 and SAP2, are induced during PTI and that these two proteases degraded the RIP pathway-associated protease MucD [52]. However, in *P*. *syringae* and *P*. *aeruginosa*, a *ΔmucD* strain has a hyper-activated AlgU phenotype [52, 53] while we have observed that PTI exposure has the opposite effect on AlgU-regulated genes. It is possible that AlgU is released via SAP1/SAP2 degradation of MucD and overactivation of the RIP cascade, but other PTI responses ultimately intervene against the AlgU regulon possibly through indirect overlapping regulators. The transcription factor AmrZ (PSPTO_1847), which was shown to have an AlgU binding site at its promoter region [11], is strongly dependent on AlgU for expression while in the plant host (Fig 6). AmrZ is one of the 14 AlgU upregulated genes that are also upregulated in PTI-activated plants compared to mock treated plants (S5 Table in S2 File), supporting the possibility that AlgU is activated during PTI exposure. However, our data showed that these 14 genes make up less than 4% of the total AlgU regulon (while in the host), whether AlgU is activated and acted against, or if AlgU activity is directly suppressed by PTI remains a question. The specific cues within the apoplast that are perceived to induce the AlgU regulon are still unknown. Determining what signals bacteria perceive in the apoplast to drive AlgU activation and whether those signals are modified or masked during PTI will be critical to understanding how this conserved bacterial sigma factor interacts with this ancient form of plant immunity.

## Material and methods

### Plant material

*Arabidopsis thaliana* Col-0 seeds were sown in SunGrow Professional growing potting mix in 3.5-inch square pots, stratified for one day at 4°C in darkness, then moved to Conviron A1000 growth chamber with settings of 14 hour day, 23°C, 70 μmol light. After two weeks, the pots were thinned to 4 plants per pot. After four weeks, the pots were moved to a growth room with settings of 12 hour day, 23°C.

### RNA sample preparation and sequencing

The same procedure as described in a previous publication was used [7]. At four to five weeks’ age, the four largest leaves of each plant were treated with flg22 or mock solution. Flg22 peptide (GenScript RP19986) stock solution was made by dissolving in DMSO to final concentration at 1mM. A 1000x dilution of the flg22 stock solution in water was syringe infiltrated to induce PTI, and a 1000x dilution of DMSO was used as the mock treatment. A 1mL blunt syringe was used to infiltrate the leaf from the abaxial side, through holes poked with a needle per half of the leaf. 24 leaves per timepoint per treatment were used. Each experiment was repeated 3 times and each produced a sequencing data set.

18–22 hours after the treatment, *Pto* was syringe-infiltrated into the treated leaves. To prepare the inoculum, *Pto* strains were first spread on King’s B Agar [54] supplemented with 20 μg/mL rifampicin and grown to lawns overnight at room temperature. Cells from the lawn were harvested and resuspended in 0.25mM MgCl2 to an optical density 600 nm of 0.8. The resuspension was used as the inoculum. After infiltration, excess liquid on the leaves were soaked up with paper towels. The bacteria strains used are listed in Table 4.

**Table 4:** Bacteria strains used.

Stock #	Genotype	Plasmid
GS_00950	*Pto* DC3000 Wild type	pJN105(empty vector)
GS_00585	*Pto* DC3000 *ΔalgUmucAB* [55]	pJN105(empty vector)

At 5 hpi, leaves were cut at the petiole-leaf blade junction. The leaves were then lined up in the middle of a sheet of parafilm. The parafilm was then folded so that the leaves are held between two layers of the parafilm with the cut side pointing at the folding line. Small openings were cut at the folding line before the assembly was rolled up from side to side and inserted into the barrel of 20-mL syringes. The syringes were put in 50mL centrifuge tubes and an RNA stabilizing buffer [56] was poured into the syringe. The tubes were then vacuumed at 96 kPA for 2 min followed by a slow release. The vacuum procedure was repeated twice to infiltrate the leaves with the buffer. The excess buffer was discarded, then the tubes with syringes inside were centrifuged at 1,000x g for 10 minutes at 4°C to collect the fluid from the apoplast. The collected fluid was then passed through 0.20-μm Micropore Express Plus membrane filters (Millipore) to collect the bacteria. The filters were then placed in homogenization tubes and frozen by liquid nitrogen before storing at −80°C.

The filters were homogenized using Geno/Grinder (SPEX SamplePrep) for 1 min at 1,750 Hz on a liquid nitrogen chilled sample holder. Trizol (Thermo Fisher Scientific) was then added, and the Direct-Zol RNA Miniprep Plus Kit (Zymo Research) or Monarch RNA isolation kit (NEB) was used for RNA extraction. An additional TURBO DNase treatment (Ambion, Invitrogen/Thermo Fisher) was carried out to further reduce DNA content, followed by a cleanup step using Monarch RNA cleanup kit (NEB). Finally, the library was produced using the TruSeq Stranded Total RNA library prep kit (Illumina) and the plant host rRNA and bacterial rRNA were depleted. RNA sample was then sent to Georgia Genomics and Bioinformatics Core for quantification, QC analysis and RNA sequencing. Single-end 75 bp reads were sequenced using Nextseq 500 system (Illumina) in high output mode.

### Data analysis

The sequencing reads were first trimmed using Trimmomatic V0.36 and read counts were computed using EDGE-pro V1.3.1 and exported using edgeToDeseq.perl. Differentially-expressed genes were then identified using DESeq2 V1.28.1, with an adjusted P value below 0.05 and a log fold change over 0.58. Venn diagram was generated with InteractiVenn website [57] and meta-chart website. Illumina sequencing data were deposited in the Gene Expression Omnibus under accession number GSE191032.

Protein function groups were manually curated by combining the outcome of Kyoto Encyclopedia of Genes and Genomes (KEGG), NCBI protein, Uniprot, pseudomonas.com, and manual curation based on literature searches. Protein cytoplasm or membrane localization was determined by checking pseudomonas.com and Uniprot.

### RT-qPCR

For real time quantification PCR analysis of selected genes, plants were treated in the same way as described above. At 5 hpi, three 0.4mm diameter leaf disks from inoculated leaves were taken and frozen by liquid nitrogen. Leaves were then crushed by Geno/Grinder in liquid nitrogen chilled sample holder. Then Trizol was added before RNA was extracted using Monarch RNA Cleanup Kit (NEB), skipping the gDNA cleaning column steps. After the extraction, the sample was treated with TURBO DNase. Monarch total RNA miniprep kit was then used to clean up the reaction. The RNA was then reverse-transcribed using QuantaBio qScript cDNA SuperMix. Luna qPCR Master Mix (NEB) was used for reaction setup, and StepOnePlus (Applied Biosystems) was used to carry out the PCR reaction. Reference genes (*hemD*, *Isc-1*, and 16s rRNA) were chosen from the list published previously [58], and showed minimal change in expression between conditions, with expression level close to genes of interest, based on the RNA-seq data. Raw data from StepOnePlus was then analyzed using LinRegPCR, and expression fold change was calculated using Microsoft Excel. Primers used are listed in Table 5.

**Table 5:** Primers used for RT-qPCR.

Gene ID^a^	Name	Forward primer	Reverse primer
PSPTO_0129	*hemD* ^b^	TCAGCAGCAGTCTGCCTTTA	GTTGCTGAACCCACACTGAA
PSPTO_1453	*lsc-1* ^b^	TCTTTGGTGGTCGTGTAATGG	GGTGTGACGCAGGTGTAATAA
multiple	*16S* ^b^	ACGGGTACTTGTACCTGGTG	CGTTTCCGAGCGTTATCCC
PSPTO_4575	*opuCA*	AACCGTCTGATCATGCCGAC	TGGATCACGTAGCCGATGTTG
PSPTO_1951	*fliD*	TGCAGGGCAAAGGCATTACC	TCGTCGAACTGAAGACCAGC
PSPTO_4001	*avrPto1*	TCCAGTCAACTGCTGAGCG	TCAGGCTTTGAGGTGCTTGG
PSPTO_1243	*algD* ^c^	GAAGAACGGCGACCTGGAACTG	CGGTGCTGCGAACCACGATAG

^a^Gene IDs are from GenBank *Pto* genome NC_004578.1.

^b^*hemD*, *lsc-1*, and 16s primers were used in Smith et al. 2018 and were used as reference genes in this study.

^c^Primer sequences of *algD* is from Markel et al. 2016.

## Supporting information

S1 File RT-qPCR confirmation of gene expression change measured from RNA-seq. Expression changes of Type III Secretion System (T3SS) structural genes. Expression changes of known *Pto* coronatine synthesis pathway genes.
(PDF)

S2 File Calculated bacterial DEGs (induced and suppressed) between different treatments. (XLSX)

## References

[1] DeFalco TA, Zipfel C. Molecular mechanisms of early plant pattern-triggered immune signaling. Molecular Cell. 2021;81: 3449–3467. doi: 10.1016/j.molcel.2021.07.029 34403694

[2] Jwa N-S, Hwang BK. Convergent Evolution of Pathogen Effectors toward Reactive Oxygen Species Signaling Networks in Plants. Front Plant Sci. 2017;8: 1687. doi: 10.3389/fpls.2017.01687 29033963 PMC5627460

[3] Ngou BPM, Jones JDG, Ding P. Plant immune networks. Trends in Plant Science. 2021; S1360138521002430. doi: 10.1016/j.tplants.2021.08.012 34548213

[4] Luna E, Pastor V, Robert J, Flors V, Mauch-Mani B, Ton J. Callose Deposition: A Multifaceted Plant Defense Response. MPMI. 2011;24: 183–193. doi: 10.1094/MPMI-07-10-0149 20955078

[5] Zipfel C, Robatzek S, Navarro L, Oakeley EJ, Jones JDG, Felix G, et al. Bacterial disease resistance in Arabidopsis through flagellin perception. Nature. 2004;428: 764–767. doi: 10.1038/nature02485 15085136

[6] Crabill E, Joe A, Block A, van Rooyen JM, Alfano JR. Plant Immunity Directly or Indirectly Restricts the Injection of Type III Effectors by the *Pseudomonas syringae* Type III Secretion System. Plant Physiology. 2010;154: 233–244. doi: 10.1104/pp.110.159723 20624999 PMC2938138

[7] Lovelace AH, Smith A, Kvitko BH. Pattern-Triggered Immunity Alters the Transcriptional Regulation of Virulence-Associated Genes and Induces the Sulfur Starvation Response in Pseudomonas syringae pv. tomato DC3000. Mol Plant Microbe Interact. 2018;31: 750–765. doi: 10.1094/MPMI-01-18-0008-R 29460676

[8] O’Malley MR, Anderson JC. Regulation of the Pseudomonas syringae Type III Secretion System by Host Environment Signals. Microorganisms. 2021;9: 1227. doi: 10.3390/microorganisms9061227 34198761 PMC8228185

[9] Nobori T, Velásquez AC, Wu J, Kvitko BH, Kremer JM, Wang Y, et al. Transcriptome landscape of a bacterial pathogen under plant immunity. Proc Natl Acad Sci USA. 2018;115: E3055–E3064. doi: 10.1073/pnas.1800529115 29531038 PMC5879711

[10] Lewis LA, Polanski K, de Torres-Zabala M, Jayaraman S, Bowden L, Moore J, et al. Transcriptional Dynamics Driving MAMP-Triggered Immunity and Pathogen Effector-Mediated Immunosuppression in Arabidopsis Leaves Following Infection with Pseudomonas syringae pv tomato DC3000. Plant Cell. 2015;27: 3038–3064. doi: 10.1105/tpc.15.00471 26566919 PMC4682296

[11] Markel E, Stodghill P, Bao Z, Myers CR, Swingle B. AlgU Controls Expression of Virulence Genes in Pseudomonas syringae pv. tomato DC3000. J Bacteriol. 2016;198: 2330–2344. doi: 10.1128/JB.00276-16 27325679 PMC4984547

[12] Ishiga T, Ishiga Y, Betsuyaku S, Nomura N. AlgU contributes to the virulence of Pseudomonas syringae pv. tomato DC3000 by regulating production of the phytotoxin coronatine. Journal of General Plant Pathology. 2018;84: 189–201. doi: 10.1007/s10327-018-0775-6

[13] Yu X, Lund SP, Greenwald JW, Records AH, Scott RA, Nettleton D, et al. Transcriptional analysis of the global regulatory networks active in Pseudomonas syringae during leaf colonization. mBio. 2014;5: e01683–01614. doi: 10.1128/mBio.01683-14 25182327 PMC4173789

[14] Wang H, Yang Z, Swingle B, Kvitko BH. AlgU, a Conserved Sigma Factor Regulating Abiotic Stress Tolerance and Promoting Virulence in Pseudomonas syringae. Mol Plant Microbe Interact. 2021;34: 326–336. doi: 10.1094/MPMI-09-20-0254-CR 33264045

[15] Chang W-S, van de Mortel M, Nielsen L, Nino de Guzman G, Li X, Halverson LJ. Alginate Production by *Pseudomonas putida* Creates a Hydrated Microenvironment and Contributes to Biofilm Architecture and Stress Tolerance under Water-Limiting Conditions. Journal of Bacteriology. 2007;189: 8290–8299. doi: 10.1128/JB.00727-07 17601783 PMC2168710

[16] Keith RC, Keith LMW, Hernández-Guzmán G, Uppalapati SR, Bender CL. Alginate gene expression by Pseudomonas syringae pv. tomato DC3000 in host and non-host plants. Microbiology. 2003;149: 1127–1138. doi: 10.1099/mic.0.26109-0 12724374

[17] Chen C, Beattie GA. Characterization of the osmoprotectant transporter OpuC from Pseudomonas syringae and demonstration that cystathionine-beta-synthase domains are required for its osmoregulatory function. J Bacteriol. 2007;189: 6901–6912. doi: 10.1128/JB.00763-07 17660277 PMC2045199

[18] Chen C, Malek AA, Wargo MJ, Hogan DA, Beattie GA. The ATP-binding cassette transporter Cbc (choline/betaine/carnitine) recruits multiple substrate-binding proteins with strong specificity for distinct quaternary ammonium compounds. Molecular Microbiology. 2010;75: 29–45. doi: 10.1111/j.1365-2958.2009.06962.x 19919675 PMC5503199

[19] Lindgren PB, Peet RC, Panopoulos NJ. Gene cluster of Pseudomonas syringae pv. “phaseolicola” controls pathogenicity of bean plants and hypersensitivity of nonhost plants. Journal of Bacteriology. 1986;168: 512–522. doi: 10.1128/jb.168.2.512-522.1986 3023280 PMC213511

[20] Cunnac S, Chakravarthy S, Kvitko BH, Russell AB, Martin GB, Collmer A. Genetic disassembly and combinatorial reassembly identify a minimal functional repertoire of type III effectors in Pseudomonas syringae. Proceedings of the National Academy of Sciences. 2011;108: 2975–2980. doi: 10.1073/pnas.1013031108 21282655 PMC3041132

[21] Wei H-L, Chakravarthy S, Mathieu J, Helmann TC, Stodghill P, Swingle B, et al. Pseudomonas syringae pv. tomato DC3000 Type III Secretion Effector Polymutants Reveal an Interplay between HopAD1 and AvrPtoB. Cell Host & Microbe. 2015;17: 752–762. doi: 10.1016/j.chom.2015.05.007 26067603 PMC4471848

[22] Worley JN, Russell AB, Wexler AG, Bronstein PA, Kvitko BH, Krasnoff SB, et al. Pseudomonas syringae pv. tomato DC3000 CmaL (PSPTO4723), a DUF1330 Family Member, Is Needed To Produce l—*allo* -Isoleucine, a Precursor for the Phytotoxin Coronatine. J Bacteriol. 2013;195: 287–296. doi: 10.1128/JB.01352-12 23144243 PMC3553850

[23] Bao Z, Wei H-L, Ma X, Swingle B. Pseudomonas syringae AlgU Downregulates Flagellin Gene Expression, Helping Evade Plant Immunity. J Bacteriol. 2020;202. doi: 10.1128/JB.00418-19 31740494 PMC6989796

[24] Dasgupta N, Wolfgang MC, Goodman AL, Arora SK, Jyot J, Lory S, et al. A four‐tiered transcriptional regulatory circuit controls flagellar biogenesis in *Pseudomonas aeruginosa*. Molecular Microbiology. 2003;50: 809–824. doi: 10.1046/j.1365-2958.2003.03740.x 14617143

[25] Nogales J, Vargas P, Farias GA, Olmedilla A, Sanjuán J, Gallegos M-T. FleQ coordinates flagellum-dependent and -independent motilities in Pseudomonas syringae pv. tomato DC3000. Appl Environ Microbiol. 2015;81: 7533–7545. doi: 10.1128/AEM.01798-15 26296726 PMC4592877

[26] Burch AY, Shimada BK, Mullin SWA, Dunlap CA, Bowman MJ, Lindow SE. Pseudomonas syringae Coordinates Production of a Motility-Enabling Surfactant with Flagellar Assembly. J Bacteriol. 2012;194: 1287–1298. doi: 10.1128/JB.06058-11 22194459 PMC3294827

[27] Taguchi F, Takeuchi K, Katoh E, Murata K, Suzuki T, Marutani M, et al. Identification of glycosylation genes and glycosylated amino acids of flagellin in Pseudomonas syringae pv. tabaci. Cellular Microbiology. 2006;8: 923–938. doi: 10.1111/j.1462-5822.2005.00674.x 16681835

[28] Liu J, Yu M, Ge Y, Tian Y, Hu B, Zhao Y. The RsmA RNA-Binding Proteins in Pseudomonas syringae Exhibit Distinct and Overlapping Roles in Modulating Virulence and Survival Under Different Nutritional Conditions. Front Plant Sci. 2021;12: 637595. doi: 10.3389/fpls.2021.637595 33719314 PMC7952654

[29] Butcher BG, Bronstein PA, Myers CR, Stodghill PV, Bolton JJ, Markel EJ, et al. Characterization of the Fur Regulon in Pseudomonas syringae pv. tomato DC3000. Journal of Bacteriology. 2011;193: 4598–4611. doi: 10.1128/JB.00340-11 21784947 PMC3165696

[30] Fishman MR, Zhang J, Bronstein PA, Stodghill P, Filiatrault MJ. Ca2+-Induced Two-Component System CvsSR Regulates the Type III Secretion System and the Extracytoplasmic Function Sigma Factor AlgU in Pseudomonas syringae pv. tomato DC3000. J Bacteriol. 2018;200. doi: 10.1128/JB.00538-17 29263098 PMC5809696

[31] Nicaise V, Joe A, Jeong B, Korneli C, Boutrot F, Westedt I, et al. Pseudomonas HopU1 modulates plant immune receptor levels by blocking the interaction of their mRNAs with GRP7. The EMBO Journal. 2013;32: 701–712. doi: 10.1038/emboj.2013.15 23395902 PMC3590987

[32] Fu ZQ, Guo M, Jeong B, Tian F, Elthon TE, Cerny RL, et al. A type III effector ADP-ribosylates RNA-binding proteins and quells plant immunity. Nature. 2007;447: 284–288. doi: 10.1038/nature05737 17450127

[33] Shimono M, Lu Y-J, Porter K, Kvitko BH, Henty-Ridilla J, Creason A, et al. The *Pseudomonas syringae* Type III Effector HopG1 Induces Actin Remodeling to Promote Symptom Development and Susceptibility during Infection. Plant Physiology. 2016;171: 2239–2255. doi: 10.1104/pp.16.01593 27217495 PMC4936540

[34] Block A, Guo M, Li G, Elowsky C, Clemente TE, Alfano JR. The Pseudomonas syringae type III effector HopG1 targets mitochondria, alters plant development and suppresses plant innate immunity. Cell Microbiol. 2010;12: 318–330. doi: 10.1111/j.1462-5822.2009.01396.x 19863557 PMC2821459

[35] Guo M, Kim P, Li G, Elowsky CG, Alfano JR. A Bacterial Effector Co-opts Calmodulin to Target the Plant Microtubule Network. Cell Host & Microbe. 2016;19: 67–78. doi: 10.1016/j.chom.2015.12.007 26764598

[36] Zhou J, Wu S, Chen X, Liu C, Sheen J, Shan L, et al. The *Pseudomonas syringae* effector HopF2 suppresses Arabidopsis immunity by targeting BAK1. The Plant Journal. 2014;77: 235–245. doi: 10.1111/tpj.12381 24237140 PMC4224013

[37] Washington EJ, Mukhtar MS, Finkel OM, Wan L, Banfield MJ, Kieber JJ, et al. *Pseudomonas syringae* type III effector HopAF1 suppresses plant immunity by targeting methionine recycling to block ethylene induction. Proceedings of the National Academy of Sciences. 2016;113: E3577–E3586. doi: 10.1073/pnas.1606322113 27274076 PMC4922156

[38] Bronstein PA, Marrichi M, Cartinhour S, Schneider DJ, DeLisa MP. Identification of a Twin-Arginine Translocation System in *Pseudomonas syringae* pv. tomato DC3000 and Its Contribution to Pathogenicity and Fitness. J Bacteriol. 2005;187: 8450–8461. doi: 10.1128/JB.187.24.8450–8461.2005 16321949 PMC1317023

[39] Charkowski A, Blanco C, Condemine G, Expert D, Franza T, Hayes C, et al. The Role of Secretion Systems and Small Molecules in Soft-Rot *Enterobacteriaceae* Pathogenicity. Annual Review of Phytopathology. 2012;50: 425–449. doi: 10.1146/annurev-phyto-081211-173013 22702350

[40] Liu L, Gueguen-Chaignon V, Gonçalves IR, Rascle C, Rigault M, Dellagi A, et al. A secreted metal-binding protein protects necrotrophic phytopathogens from reactive oxygen species. Nature Communications. 2019;10. doi: 10.1038/s41467-019-12826-x 31649262 PMC6813330

[41] Yamazaki A, Li J, Hutchins WC, Wang L, Ma J, Ibekwe AM, et al. Commensal Effect of Pectate Lyases Secreted from *Dickeya dadantii* on Proliferation of *Escherichia coli* O157:H7 EDL933 on Lettuce Leaves. Applied and Environmental Microbiology. 2011;77: 156–162. doi: 10.1128/AEM.01079-10 21075884 PMC3019694

[42] Teixeira PJPL Colaianni NR, Law TF Conway JM, Gilbert S, Li H, et al. Specific modulation of the root immune system by a community of commensal bacteria. Proceedings of the National Academy of Sciences. 2021;118: e2100678118. doi: 10.1073/pnas.2100678118 33879573 PMC8072228

[43] Lewenza S, Charron-Mazenod L, Afroj S, van Tilburg Bernardes E. Hyperbiofilm phenotype of Pseudomonas aeruginosa defective for the PlcB and PlcN secreted phospholipases. Can J Microbiol. 2017;63: 780–787. doi: 10.1139/cjm-2017-0244 28609638

[44] Swietnicki W, Czarny A, Antkowiak L, Zaczynska E, Kolodziejczak M, Sycz J, et al. Identification of a potent inhibitor of type II secretion system from Pseudomonas aeruginosa. Biochem Biophys Res Commun. 2019;513: 688–693. doi: 10.1016/j.bbrc.2019.04.055 30987825

[45] Naskar S, Hohl M, Tassinari M, Low HH. The structure and mechanism of the bacterial type II secretion system. Molecular Microbiology. 2021;115: 412–424. doi: 10.1111/mmi.14664 33283907

[46] Pineau C, Guschinskaya N, Robert X, Gouet P, Ballut L, Shevchik VE. Substrate recognition by the bacterial type II secretion system: more than a simple interaction. Mol Microbiol. 2014;94: 126–140. doi: 10.1111/mmi.12744 25098941

[47] Tilley D, Law R, Warren S, Samis JA, Kumar A. CpaA a novel protease from Acinetobacter baumannii clinical isolates deregulates blood coagulation. FEMS Microbiol Lett. 2014;356: 53–61. doi: 10.1111/1574-6968.12496 24910020

[48] Urusova DV, Kinsella RL, Salinas ND, Haurat MF, Feldman MF, Tolia NH. The structure of Acinetobacter-secreted protease CpaA complexed with its chaperone CpaB reveals a novel mode of a T2SS chaperone-substrate interaction. J Biol Chem. 2019;294: 13344–13354. doi: 10.1074/jbc.RA119.009805 31320476 PMC6737226

[49] Putker F, Tommassen-van Boxtel R, Stork M, Rodríguez-Herva JJ, Koster M, Tommassen J. The type II secretion system (Xcp) of Pseudomonas putida is active and involved in the secretion of phosphatases. Environ Microbiol. 2013;15: 2658–2671. doi: 10.1111/1462-2920.12115 23530902

[50] Sarris PF, Skandalis N, Kokkinidis M, Panopoulos NJ. In silico analysis reveals multiple putative type VI secretion systems and effector proteins in Pseudomonas syringae pathovars: Putative T6SS in P. syringae pathovars. Molecular Plant Pathology. 2010; no-no. doi: 10.1111/j.1364-3703.2010.00644.x 21091602 PMC6640432

[51] Helmann TC, Deutschbauer AM, Lindow SE. Genome-wide identification of Pseudomonas syringae genes required for fitness during colonization of the leaf surface and apoplast. Proc Natl Acad Sci U S A. 2019;116: 18900–18910. doi: 10.1073/pnas.1908858116 31484768 PMC6754560

[52] Wang Y, Garrido-Oter R, Wu J, Winkelmüller TM, Agler M, Colby T, et al. Site-specific cleavage of bacterial MucD by secreted proteases mediates antibacterial resistance in Arabidopsis. Nat Commun. 2019;10: 2853. doi: 10.1038/s41467-019-10793-x 31253808 PMC6599210

[53] Damron FH, Yu HD. Pseudomonas aeruginosa MucD regulates the alginate pathway through activation of MucA degradation via MucP proteolytic activity. J Bacteriol. 2011;193: 286–291. doi: 10.1128/JB.01132-10 21036998 PMC3019965

[54] King EO, Ward MK, Raney DE. Two simple media for the demonstration of pyocyanin and fluorescin. J Lab Clin Med. 1954;44: 301–307. 13184240

[55] Park SH, Bao Z, Butcher BG, D’Amico K, Xu Y, Stodghill P, et al. Analysis of the small RNA spf in the plant pathogen Pseudomonas syringae pv. tomato strain DC3000. Microbiology (Reading). 2014;160: 941–953. doi: 10.1099/mic.0.076497-0 24600027

[56] Wit P, Pespeni MH, Ladner JT, Barshis DJ, Seneca F, Jaris H, et al. The simple fool’s guide to population genomics via RNA ‐Seq: an introduction to high‐throughput sequencing data analysis. Molecular Ecology Resources. 2012;12: 1058–1067. doi: 10.1111/1755-0998.12003 22931062

[57] Heberle H, Meirelles GV, da Silva FR, Telles GP, Minghim R. InteractiVenn: a web-based tool for the analysis of sets through Venn diagrams. BMC Bioinformatics. 2015;16. doi: 10.1186/s12859-015-0611-3 25994840 PMC4455604

[58] Smith A, Lovelace AH, Kvitko BH. Validation of RT-qPCR Approaches to Monitor *Pseudomonas syringae* Gene Expression During Infection and Exposure to Pattern-Triggered Immunity. Molecular Plant-Microbe Interactions®. 2018;31: 410–419. doi: 10.1094/MPMI-11-17-0270-TA 29436925

