# Supplementary material for: In planta transcriptomics reveals conflicts between pattern-triggered immunity and the AlgU sigma factor regulon
Source: PLoS One. 2022 Sep 1;17(9):e0274009. doi: 10.1371/journal.pone.0274009 (PMC9436044; doi:10.1371/journal.pone.0274009)
Supplement: S1 File — Expression changes of Type III Secretion System (T3SS) structural genes. Expression changes of known Pto coronatine synthesis pathway genes. (PDF) [file pone.0274009.s001.pdf]

Figure S1

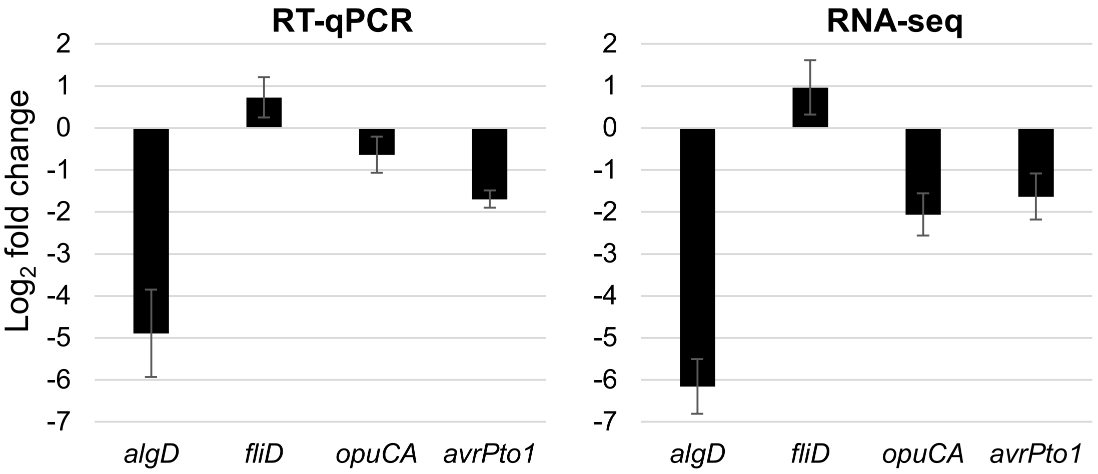

Figure S1. Left: Reverse Transcription-quantitative PCR (RT-qPCR) measured expression change in *ΔalgU* compared to wild type background at 5 hpi in naïve leaves. 16s rRNA was used for standardizing bacterial template during PCR setup, and *hemD* and *lsc-1* were used as reference genes. Right: RNA-sequencing measured expression changes from the same comparison. Error bar shows standard deviation from three replicated experiments.

Figure S2

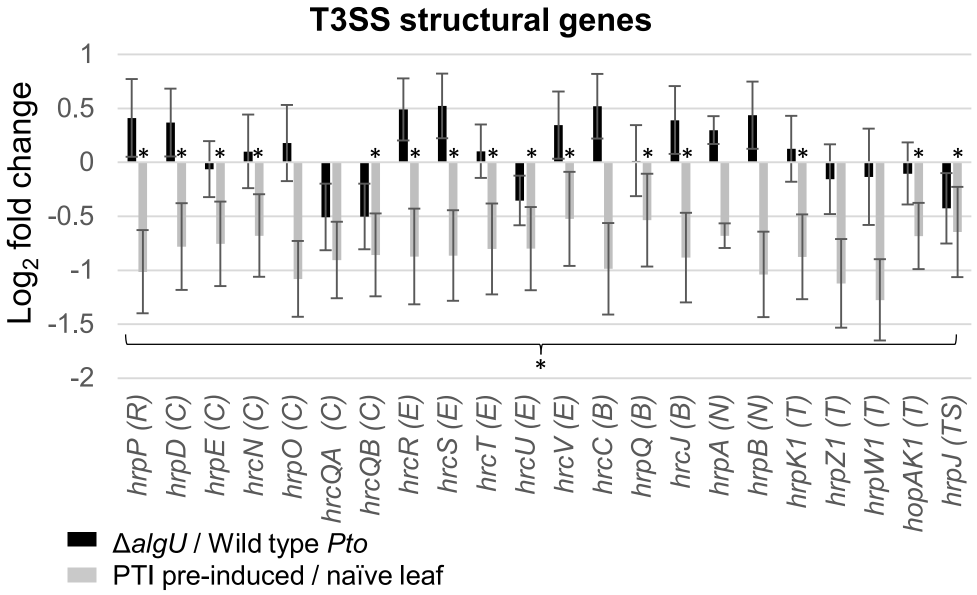

Figure S2. Expression changes of Type III Secretion System (T3SS) structural genes. Genes are arranged in spatial order based on T3SS structure. R: regulator; C: cytoplasmic complex; E: export apparatus; B: basal body; N: needle; T: translocator; TS: translocator switch. * indicates genes with padj >0.05 calculated by DESeq2. * for black bars are below the line of 0, * for grey bars are above the line of 0.

Figure S3

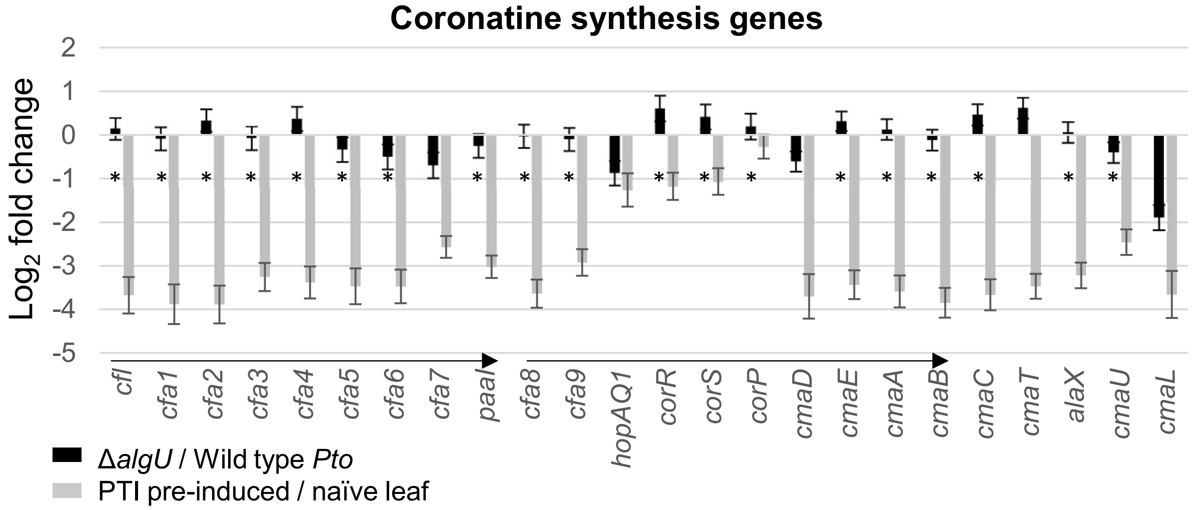

Figure S3. Expression changes of known *Pto* coronatine synthesis pathway genes. * indicates genes with padj >0.05 calculated by DESeq2. All * in this graph are from black bars. Arrows indicate genes within an operon.
